# Targeted Regulation of Nuclear Lamins by Ubiquitin and Ubiquitin-Like Modifiers

**DOI:** 10.3390/cells9061340

**Published:** 2020-05-27

**Authors:** Michael Blank

**Affiliations:** Laboratory of Molecular and Cellular Cancer Biology, Azrieli Faculty of Medicine, Bar-Ilan University, Safed 1311502, Israel; michael.blank@biu.ac.il

**Keywords:** nuclear lamins, laminopathies, ubiquitin, ubiquitin-like modifiers, autophagy, proteasome

## Abstract

Nuclear lamins (NLs) are essential components of the animal cell nucleus involved in the regulation of a plethora of molecular and cellular processes. These include the nuclear envelope assembly and stability, mechanotransduction and chromatin organization, transcription, DNA replication, damage repair, and genomic integrity maintenance. Mutations in NLs can lead to the development of a wide range of distinct disease phenotypes, laminopathies, consisting of cardiac, neuromuscular, metabolic and premature aging syndromes. In addition, alterations in the expression of nuclear lamins were associated with different types of neoplastic diseases. Despite the importance and critical roles that NLs play in the diverse cellular activities, we only recently started to uncover the complexity of regulatory mechanisms governing their expression, localization and functions. This integrative review summarizes and discusses the recent findings on the emerging roles of ubiquitin and ubiquitin-like modifiers (ULMs) in the regulation of NLs, highlighting the intriguing molecular associations and cross-talks occurring between NLs and these regulatory molecules under physiological conditions and in the disease states.

## 1. Nuclear Lamins and Associated Disorders: An Overview

NLs are the major structural components of the nuclear lamina. They are situated between the peripheral heterochromatin and inner nuclear membrane (INM), and are found in all multicellular metazoans of the animal kingdom. These type V intermediate filament (IF) proteins together with the lamina-associated factors form a dense protein meshwork endowing the nucleus with its shape and mechanical stability. Additionally, serving as a binding platform for proteins and chromatin domains, NLs are implicated in key nuclear activities, including higher-order chromatin organization, transcription, DNA replication, and damage repair [1,2,3,4]. Remarkably, in addition to their primary localization beneath the INM, some types of lamins (i.e., A-type lamins) are found in the nuclear interior, forming discrete foci or a diffuse network. This intranuclear pool of lamins has been suggested to play an important role in spatial chromatin organization and accessibility through the dynamic binding of hetero- and euchromatic genomic regions and promoter subdomains, thereby affecting gene expression [5].

Based on a protein structure, biochemical properties and expression pattern, NLs are subdivided into two subtypes: A-type lamins (A-lamins), which are developmentally regulated and primarily expressed in differentiated cells, and B-type lamins (B-lamins), which are constitutively expressed in all somatic cells. The A-type lamins, which include lamin A, C, C2 and lamin AΔ10, are encoded by a single gene (*LMNA* in humans). Lamin A and lamin C are the major A-type lamins expressed in somatic cells. Two minor isoforms of A-lamins, lamin AΔ10 and C2, were found to be expressed either in somatic cells (AΔ10) or in germ cells (C2), but their functions and regulations are poorly understood [6,7]. In contrast to the A-type lamins, B-lamins, lamin B1, and lamin B2, are encoded by two separate genes *LMNB1* and *LMNB2*. A testis-specific lamin B3, derived from *LMNB2* by alternative splicing, has also been reported [8].

**Figure 1:**
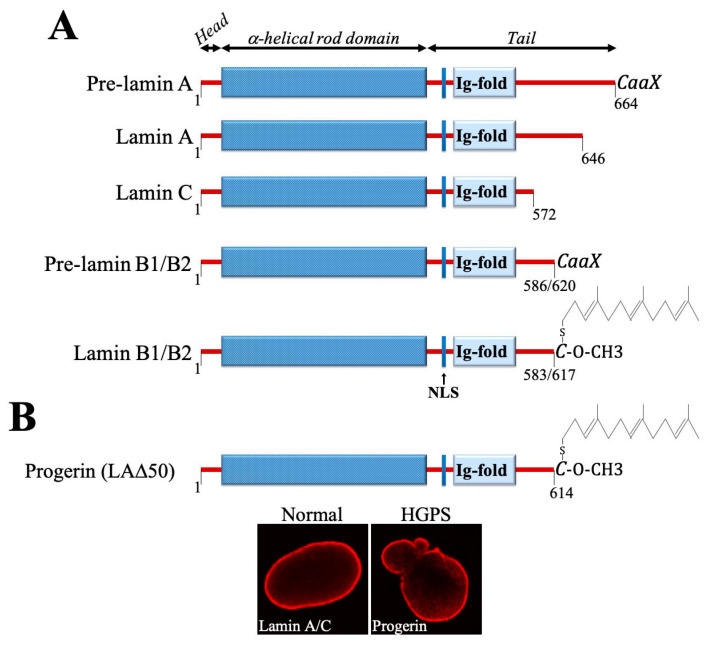
(**A**) Schematic diagram of domain composition and the presence of CaaX motif in nuclear lamins. NLS, nuclear localization signal. (**B**) Progerin, a mutant form of lamin A, lacks 50 amino acids in the C-terminus and therefore cannot be properly processed, retaining the carboxyl-terminal farnesyl-cysteine-methyl-ester. The morphological view of normal and HGPS cell nuclei is shown on the bottom. The images derived from [10].

Both A- and B-lamins share a similar domain organization with other IF proteins and incorporate in their structure an N-terminal head domain, a central coiled-coil rod domain and a C-terminal tail domain (Figure 1A). In addition, they contain a nuclear localization signal (NLS) and an immunoglobulin (Ig) fold. Furthermore, lamin A (pre-lamin A) and lamins B1 and B2—but not lamin C—have a carboxy-terminal CaaX box/motif (C, cysteine; a, an aliphatic amino acid; X, any amino acid), whose posttranslational modifications (PTMs) are essential for the correct incorporation of these proteins into the nuclear lamina. The cysteine residue in the CaaX motif undergoes several sequential PTMs, including farnesylation and methyl esterification. Pre-lamin A is further processed through the proteolytic cleavage by ZMPSTE24 protease that removes the modified cysteine together with the 14 other amino acids to generate the mature A-lamin. In contrast, B-lamins remain permanently farnesylated and carboxy-methylated (Figure 1A). It is thought that farnesylation enhances the association of B-lamins with the INM, while the lack of this modifications in A-lamins render them to be more soluble and loosely associated with the nuclear envelope (NE), contributing to the formation of lamina-independent nucleoplasmic pool [4,5,7,9]. To note, A-lamins are undetectable in the nuclear interior of non-cycling, quiescent, terminally-differentiated and senescent cells, suggesting a link between their nucleoplasmic sequestration and cell cycling [5].

Accumulating evidence suggests that A- and B-type lamins form separate meshworks and have relatively non-redundant functions. Interestingly, while B-lamins have been shown to contribute to the nuclear elasticity, the expression of A-lamins affects the nuclear stiffness, defining the deformability of the nucleus and the ability of migrating cells to move through a confined space with the narrow constrictions (e.g., during metastatic dissemination) [11,12,13,14].

To date, several hundred mutations, particularly in the *LMNA* gene, have been associated with a diverse spectrum of human disorders, collectively known as laminopathies, whose clinical manifestations include cardiac and muscular dystrophy, lipodystrophy, leukodystrophy, dermopathy, neuropathy, dysplasia, as well as premature aging syndromes [7,15]. The disease-causing mutations in *LMNA*, leading to primary laminopathies, can affect the functions of both peripheral and the nucleoplasmic pool of lamin A, and/or change their respective localization [5]. Secondary laminopathies, which are caused by mutations in the *LMNB1* or *LMNB2* genes, *ZMPSTE24* or in genes encoding for lamin-binding proteins (e.g., *EMD*/emerin) were also described [15]. To note, many mutations in NL genes are autosomal dominant, and laminopathies are rare diseases [9]. 

One of the best studied and most severe laminopathies is the Hutchinson-Gilford progeria syndrome (HGPS). This fatal genetic condition, characterized by accelerated/premature aging that typically develops in childhood, mostly arises due to the heterozygous silent mutation in exon 11 of *LMNA* (c.1824C>T, p.G608G). This silent mutation activates a cryptic donor splice site leading to the deletion of 50 amino acids near the C-terminus of pre-lamin A and the production of a permanently farnesylated and carboxymethylated dominant protein known as progerin (Figure 1B) [16,17]. Progerin expression entails severe cellular defects, affecting nuclear morphology and nucleoplasmic transport, chromatin organization and DNA repair, disrupts telomeres, redox homeostasis, causes genome instability, and leads to premature senescence [7,18,19,20]. Remarkably, progerin was also shown to accumulate in normal cells during physiological aging and, supposedly, in cancer, where it could promote genomic instability and increase tumorigenesis [21,22,23,24]. Interestingly, genomic studies showed that *LMNA* is not frequently altered in human malignancies, however changes in its expression are common in many cancers [12,14,25,26,27,28,29,30,31,32,33,34,35,36,37,38,39]. Dysregulated expression of *LMNB1* was also associated with several types of neoplastic diseases and patient survival. The studies showed that lamin B1 is overexpressed in hepatocellular carcinoma (HCC) and positively correlates with tumor stage, size and number of tumor nodules in HCC patients [40]. Similarly, the elevated levels of lamin B1 were reported in pancreatic cancer and associated with an increased incidence of distant metastases and poor patient prognosis [41]. In contrast, in lung cancer and some hematological malignancies (i.e., B-cell lymphomas) lamin B1 expression is significantly diminished, and was suggested as an adverse prognostic factor [42,43].

## 2. Ubiquitin, Ubiquitin-Like Modifiers (ULMs), and the Conjugation Pathways

**Figure 2:**
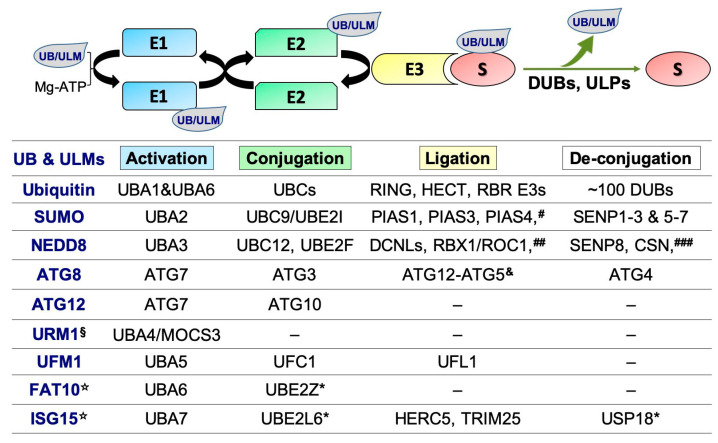
Figure **2.** The ubiquitin and ULM conjugation cascade/s and the regulatory enzymes. * These enzymes also work in the ubiquitin pathway [47,50]. **^#^** In addition to the listed E3s, several other proteins were reported to function as SUMO E3 ligases, including RanBP2, ZNF451, TRIM28/KAP1, PML, and the polycomb protein Pc2 [51,52]. To note, in order to be conjugated (SUMOylated) to their cellular targets by the E1-E2-E3 cascade, SUMO proteins (SUMO1, SUMO2 and SUMO3) should first be terminally processed from their corresponding precursor forms by SUMO-specific cysteine proteases (SENPs). This processing exposes two C-terminal glycine residues of SUMO proteins and generates their mature forms which can be bound by E1. Essentially, SENPs also possess isopeptidase activity, which is critical for de-conjugation of SUMO proteins from their substrates and SUMO recycling [53]. **^##^** Other E3s which have been reported to promote NEDD8 conjugation (neddylation) include RBX2/ROC2/SAG, MDM2, c-CBL, Parkin, IAPs, RNF111, TRIM40, and SCF^FBXO11^ [54]. **^###^** The deneddylases which could also remove NEDD8 from the modified proteins include Ataxin-3, USP21, UCH-L1 and UCH-L3 [55]. **^&^** The Atg12-Atg5 functions as an E3, promoting the conjugations of phosphatidylethanolamine (PE) to ATG8. The lipidated ATG8 (ATG8-PE) plays essential roles in autophagosome formation and selective cargo recognition during autophagy. It is recycled by ATG4 protease [56,57,58]. **^§^** Ubiquitin-like modifier URM1 acts as a sulphur carrier in the process of eukaryotic transfer RNA (tRNA) modification [59,60]. **^☆^** In contrast to ubiquitin and other ULMs, ISG15 and FAT10 comprises two ubiquitin-like domains joined by a flexible linker [61]. There is accumulating evidence showing that ubiquitin and ULMs could generate hybrid chains, which seem to confer improved specificity and affinity towards their cognate receptors and to diversify the “ubiquitin code” [62].

Ubiquitin and ULMs, which in humans consist of eight members of structurally-related to ubiquitin molecules (i.e., SUMOs, NEDD8, ATG8, ATG12, URM1, UFM1, FAT10 and ISG15) (Figure 2), play fundamental roles in a wide range of biological processes by regulating protein stability, localization, interactions, and function/s [44,45,46,47].

In humans, there are at least four genes encoding for SUMO proteins (*SUMO1-4*) and seven for ATG8 (*MAP1LC3A*, *MAP1LC3B*, *MAP1LC3B2*, *MAP1LC3C*, *GABARAP*, *GABARAPL1* and *GABARAPL2*). Ubiquitin is encoded by four different genes *UBB*, *UBC*, *UBA52* and *RSP27A*. Expression of *UBB* and *UBC* generate three- and nine-repeats poly-ubiquitin precursors, respectively. These precursors are subsequently processed by deubiquitinating enzymes (DUBs) to generate mature free ubiquitin molecules which are then used in the ubiquitination cascade. *UBA52* and *RSP27A* code for a single-copy ubiquitin fused to the ribosomal proteins L40 and S27A, respectively.

Ubiquitin and most ULMs are conjugated to their cellular targets via their C-terminal di-glycine motif during a highly-orchestrated multi-step enzymatic reaction mediated by three principal types of enzymes: E1, E2, and E3s (Figure 2). The conjugation of ubiquitin to the designated substrates involves: (1) ubiquitin-activating enzymes (E1s), which bind to and activate ubiquitin molecules using the energy of ATP hydrolysis (two E1s were found in humans: UBA1 and UBA6); (2) ubiquitin-conjugating enzymes (E2s, also referred to as ubiquitin carrier proteins or UBCs, ~40 members), which accept ubiquitin from E1; and 3) ubiquitin E3 ligases that recruit ubiquitin-charged E2s and mediate ubiquitin transfer to the substrate (~650 E3s). Depending on the domain characteristics and ubiquitin transfer mechanism, E3s are classified into one of E3 ubiquitin ligase families: really interesting new gene (RING; ~600 members), homologous to the E6AP carboxyl terminus (HECT) domain E3s (~30 members), and RING-in-between-RING (RBR) E3s, a unique family of hybrid RING-HECT type E3s (~12 in humans) [48,49]. ULMs are also conjugated to their substrates through a mechanism similar to the ubiquitination cascade. Modifications of protein targets by ubiquitin and ULMs are mostly reversible through the action of specific enzymes, DUBs and ULPs [44,50,51,52,53,54,55,56,57,58]. Due to the intrinsic involvement of ubiquitination and ULM-conjugation/deconjugation pathways in the regulation of virtually every biological process occurring in the cell, their components are under extensive investigations as disease biomarkers and drug targets for a variety of human disorders, including cancer.

## 3. Regulation of NLs by the Ubiquitin and ATG8-ATG12 Conjugation Systems

**Figure 3:**
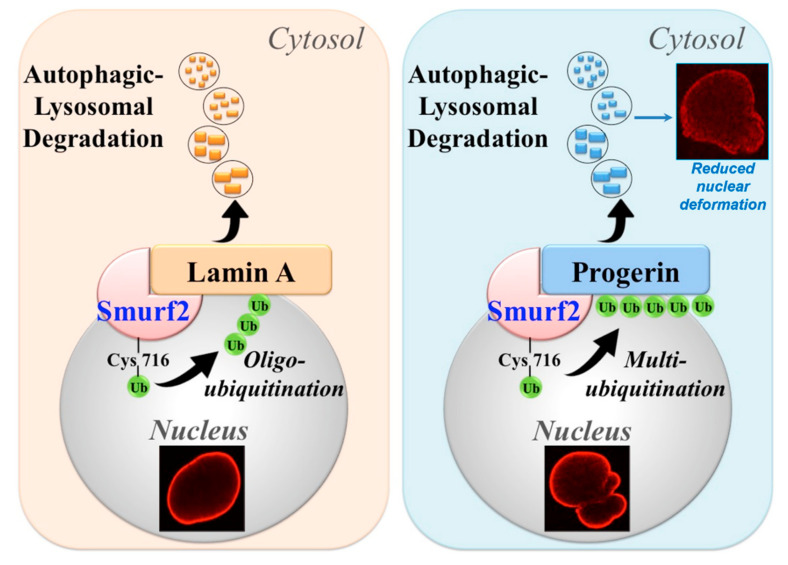
SMURF2 regulates stability and the autophagic-lysosomal turnover of lamin A and progerin. SMURF2 was shown to oligo-ubiquitinate lamin A and multi-monoubiquitinate progerin, targeting these proteins for degradation through autophagy. Cys716, the active-site cysteine of SMURF2. The confocal images presented in the figure are from [10].

Recently, we reported that protein expression and stability of lamin A and progerin are regulated by the HECT-type E3 ubiquitin ligase and suggested tumor suppressor, SMURF2 [10,63,64,65,66]. We demonstrated that SMURF2 directly binds, ubiquitinates, and negatively regulates the expression levels of lamin A and progerin in SMURF2 dose- and E3 ligase-dependent manners, promoting the autophagic-lysosomal degradation of these proteins (Figure 3). Interestingly, the data showed that SMURF2 ubiquitinates lamin A and progerin in a distinctive manner: lamin A was mostly oligo-ubiquitinated by SMURF2, while progerin was found to undergo multi-monoubiquitination. Remarkably, the results obtained in human dermal fibroblasts (HDFs), derived from healthy or HGPS patients, indicated that the adventitious expression of SMURF2 decreases progerin levels twice as efficiently than of lamin A. This phenomenon was associated with a significantly reduced nuclear deformability in HGPS cells (Figure 3) [10]. Finally, the immunohistochemical studies conducted on tissue samples of *Smurf2* knock-out vs. wild-type mice, as well as on human tissue microarrays, provided additional evidence that SMURF2 operates as a bona fide regulator of A-lamins. Collectively, these findings suggested SMURF2 as a key regulator of lamin A and progerin, fine-tuning their expression levels through the ubiquitin-mediated autophagic turnover. Future studies should determine the specific residues serving as ubiquitin acceptors on these proteins and examine the consequences of their alterations on the localization, assembly and dynamics of NLs. Additionally, it would be important to determine the linkage type of oligo-ubiquitination installed on lamin A by SMURF2, as well as to examine whether and how the distinctive ubiquitination patterns of lamin A and progerin are linked to their differential degradation rate. It would be also interesting to investigate whether and how SMURF2 and its closely-related family members, NEDD4 E3s [67], affect the expression and functions of other nuclear factors, which alterations lead to laminopathic phenotypes.

A-lamins were reported to interact with the components of autophagic machinery and undergo degradation in DNA-damaged cells as well. Li et al. showed that following cell treatment with doxorubicin, a DNA intercalating drug and double-strand breaks inducer, the levels of lamin A/C, but not lamin B1 or B2, were significantly decreased [68]. They also showed that this treatment triggered DNA leakage, activating the selective degradation of nuclear constituents through the autophagy/nucleophagy. Mechanistically, they demonstrated that DNA damage induced the nuclear accumulation of SUMO-conjugating enzyme Ubc9 (UBE2I in humans), leading to SUMOylation of lamin A/C. This modification was required for lamin A/C interaction with the autophagy-related ubiquitin-like protein ATG8/LC3. Knockdown of Ubc9 attenuated the SUMOylation of lamin A/C and markedly reduced nucleophagy.

The expression levels of B-lamins were also shown to be regulated by the autophagic machinery. Berger’s group discovered that while ATG8-lamin B1 interactions occur in cells at the basal level, they do not initiate lamin B1 degradation. However, following oncogenic and genotoxic stress lamin B1 is efficiently targeted for the autophagic breakdown, driving cellular senescence to restrain cell proliferation and oncogenesis [69]. Intriguingly, Peeper’s group showed that cells entering oncogene-induced senescence downregulate not only lamin B1 but also lamin A, as well as levels of several other NE proteins, resulting in an altered NE morphology and disrupted nuclear structure [70]. The authors also provided evidence that depletion of either *LMNB1* or *LMNA* can, at least partially, recapitulate the senescence phenotype and NE defects induced by oncogenic stress. Finally, they demonstrated that the global loss of NE proteins, including lamin A and B1, is a consequence of their enhanced autophagic turnover.

Lamin B1 was also reported to undergo proteasomal degradation, mediated by different types of E3s. Parnaik’s group showed that RING-finger E3 ubiquitin ligase RNF123 promotes proteasomal turnover of lamin B1 and several lamin-binding proteins, including lamina-associated polypeptide 2α (LAP2α), emerin and pRb [71]. Interestingly, the authors found that expression of lamin A rod-domain mutants associated with the Emery-Dreifuss muscular dystrophy (EDMD), G232E, Q294P and R386K, considerably increased the expression of RNF123, which was associated with decreased protein levels of lamin B1, LAP2α and pRb. The same group also showed that HECT-type E3 ligase and SMURF2 family member HECW2 ubiquitinates and targets lamin B1 for proteasomal proteolysis, and that levels of HECW2 are upregulated in cells expressing the EDMD mutants [72].

Recently, Zhen and colleagues reported that during terminal erythropoiesis, which is characterized by erythroblast nuclear condensation and enucleation, Wdr26, a core component of the CTLH E3 ubiquitin ligase complex, targets several nuclear proteins, including B-lamins, for ubiquitination and proteasomal degradation [73]. Interestingly, the expression levels of lamin A/C were mostly unaffected by Wdr26 or by depletion of Rmnd5a, another core component of the CTLH E3 ligase complex which cooperates with Wdr26 in the regulation of B-lamins. The failure of the CTLH complex to degrade lamin B in differentiating erythroblasts blocked nuclear opening formation and delayed nuclear condensation.

Lastly, in the study just published by Boisvert’s group the authors reported that several ubiquitin pseudogenes, including UBB pseudogene 4 (*UBBP4*), which encodes UB^KEKS^ (Q2K, K33E, Q49K, N60S), are expressed in cells, similar to other genuine protein-coding genes. Intriguingly, their data pointed out that these genes are functionally different from canonical ubiquitin (UB): they do attach to different protein targets, including NLs (i.e., lamin A and lamin B1/B2), but do not target them for proteasomal degradation [74]. Instead, the authors showed that expression of UB^KEKS^ is important for a proper subcellular localization of NLs and cell growth: cells knocked-out for *UBBP4* exhibited marked accumulation of lamin A within nucleoli and were delayed in growth. Their results also suggested that NLs could be specifically targeted for modification by UB^KEKS^. The findings showed that although lamin A and B2 were mostly modified by ubiquitin (vs. UB^KEKS^; at ratio 20:1), the assessed amount of UB^KEKS^ conjugated to nuclear lamins was much higher when compared to the UB:UB^KEKS^ expression ratio (700:1). The enzymatic components responsible for modifications of NLs by UB^KEKS^ vs. canonical ubiquitin are awaiting to be determined in the future studies.

## 4. Modifications of NLs by SUMO

One of the first indications on the association between nuclear lamins and the SUMOylation cascade was provided by Brown’s group. Using a yeast two-hybrid system and co-immunoprecipitations, they found that lamin A/C and progerin interact with the SUMO-conjugating enzyme Ubc9 and Mel-18/PCGF2—the suggested anti-SUMO E3 that inhibits the ability of Ubc9 to transfer SUMO to the target protein/s [75]. Later investigations, conducted by Zhang and Sarge, revealed that lamin A is indeed SUMOylated on K201 residue. This residue is located within the SUMOylation consensus sequence ΨKXE in the lamin A coiled-coil rod-domain (M_200_K_201_E_202_E_203_) [76]. Their data also showed that lamin A is more efficiently modified by SUMO2 than by SUMO1. Subsequently, Galisson and colleagues demonstrated that the K420 site, which is located within the NLS of lamin A, is SUMOylated by SUMO3 as well [77]. To note, SUMO3 shares 97% amino acid identity with SUMO2, and it is ~50% identical to SUMO1 [51]. Additionally, the data obtained by Zhang and Sarge [76] showed that two lamin A mutants associated with familial dilated cardiomyopathy (DCM) and conduction system disease, E203G and E203K, exhibit decreased SUMOylation. They further showed that K201R, E203G, and E203K have a similar aberrant localization pattern, concentrating into discrete nuclear foci. This phenomenon was monitored both in mouse embryonic cardiomyocytes and in HeLa cells. Moreover, they demonstrated that overexpression of these mutants in HeLa cells leads to increased cellular demise. These associations suggest that aberrant SUMOylation of lamin A can be a cause of at least some laminopathic phenotypes.

Interestingly, the results obtained by Hirose’s group showed that although K201R and E203G *LMNA* mutations resulted in defective SUMOylation of the lamin A polypeptide, as shown in the previous study, these mutants had a different sub-nuclear localization: a large portion of the E203G mutant was distributed inside the nuclei, whereas K201R lamin A exhibited normal localization [78]. Moreover, the authors showed that E203G, but not SUMO2-insensitive K201R lamin A, had a dominant-negative effect on wild-type protein, and its low-dose/long-term expression can induce the premature senescence in HDF cells. Based on these results, the authors concluded that defective SUMOylation of lamin A on K201 is not a predominant cause of familial dilated cardiomyopathy. Noteworthy, Hirose’s group also reported that lamin A interacts with SUMO2 via one of its four SUMO-interacting motifs (SIM3) residing in the Ig-fold of A-lamin. Additionally, they demonstrated that SIM3 is involved in lamin A chromosomal accumulation during telophase, and that interactions between SIM3 of lamin A and a putative SUMO2-modified protein plays an important role in the reorganization of the nuclear lamina at the end of mitosis [79].

The tail domain of lamin A was also shown to be modified by SUMO. Wilson’s group reported that in addition to K420 residue, which was previously mapped as lamin A SUMOylation site [77], the K486 residue of lamin A Ig-fold could also be modified by SUMO1 [80]. Remarkably, the authors demonstrated that mutations associated with familial partial lipodystrophy (FPLD; a laminopathy associated with a loss of subcutaneous adipose tissue), including G465D and K486N, markedly reduce SUMOylation of the lamin A tail. Since K420 and K486 residues are present in both lamin C and progerin and can also be modified by ubiquitin [81] and, potentially, by other ULMs, it will be interesting to determine the interplay between these PTMs and the downstream functions of these lamins. Noteworthy, SUMO1-dependent conjugation of lamin A/C was shown to be modulated by the SUMO protease SENP1 and required for lamin A/C interaction with pRb [82]. The authors also showed that SUMOylation of both lamin A/C and pRb protected these proteins from proteasomal degradation, underscoring the significance of this modification in the regulation of NLs and their binding partners.

## 5. Effects of Laminopathic Mutations on SUMO and the SUMOylation Pathway

Laminopathic mutations were also reported to affect the localization and functions of the SUMOylation machinery. Tesson’s group showed that overexpression of mutant lamin C (D192G), which is also associated with DCM, results in the mis-localization and trapping of SUMO1 proteins inside lamin C nuclear aggregates, the phenomenon that was abrogated by the disruption of the SUMO1 di-glycine motif required for SUMOylation [83]. Subsequently, Tesson’s group showed that overexpression of other disease-associated lamin A/C mutants, including DCM-related Q353K and EDMD-related H222P and R386K mutants, also affects the localization of SUMO1 and Ubc9 in transfected mouse myoblast C2C12 cells [84]. Remarkably, the authors presented evidence for the altered expression of SUMO1 in muscle tissues of *Lmna*^H222P/H222P^ knock-in mice. These mice develop adult-onset muscle dystrophy and DCM comparable to the human disease. Interestingly, their data also showed that lamin A/C itself was not modified by SUMO1, suggesting that targeting A-lamins by this modifier depends on particular cellular settings/context. Cumulatively, the obtained results suggested that by modifying the nuclear pool of SUMO and SUMOylated proteins *LMNA* mutations may disturb normal cellular functions regulated by this pathway. These functions include chromatin organization, DNA repair and transcription regulation [51]. Alterations in these vital processes are closely-associated with laminopathic phenotypes and might be aggravated by dysfunctional SUMOylation and improperly modified nuclear proteins.

Progerin has also been reported to affect the SUMOylation. Kelley and colleagues showed that progerin expression significantly reduces the nuclear levels of Ubc9 and SUMO2/3. This phenomenon was correlated with a disruption of the Ran gradient, the nuclear localization of the nucleoporin TPR and reduced trimethylation of histone H3 on K9 (H3K9me3) [85]. Moreover, the authors showed that forced nuclear expression of Ubc9 in progerin-expressing cells can both rescue the Ran gradient and Ran-dependent import of TPR, and restore the nuclear levels of SUMO2/3. Their data also suggested that the release from chromatin of the Ran nucleotide exchange factor RCC1 is regulated by SUMOylation, and that defects in this pathway might play a pathogenic role in HGPS. 

## 6. Conclusions

A mounting body of evidence suggests that ubiquitin and its structurally-related family members play pivotal roles in the regulation of nuclear lamins. The obtained results show that modifications of NLs by ubiquitin and ULMs are important not just for their stability regulation, but also for coordinating their subcellular localization, dynamics, molecular interactions and functions. Further investigations aimed to unravel the detailed mechanisms underlying nuclear lamina regulation by UB/ULM-conjugation pathways and the elucidation of molecular cross-talks between these and other PTMs regulating nuclear lamins (e.g., phosphorylation, oxidation, *O*-GlcNAcylation, acetylation [86]) could illuminate novel opportunities for therapeutic interventions in the diverse spectrum of human disorders associated with lamin dysfunction.

## Abbreviations

ATG—autophagy-related gene
c-CBL—casitas B-lineage lymphoma
CSN—COP9 (constitutive photomorphogenesis 9) signalosome
CTLH—C-terminal to LisH complex
DCM—dilated cardiomyopathy
DCN1—defective in cullin neddylation 1 protein
DCNLs—DCN1-like proteins
DSM—familial dilated cardiomyopathy
DUBs—deubiquitinases
EDMD—Emery-Dreifuss muscular dystrophy
EFP—estrogen-responsive finger protein
EMT—epithelial-to-mesenchymal transition
GABARAP—gamma-aminobutyric acid receptor-associated protein
GABARAPL1—gamma-aminobutyric acid receptor-associated protein like 1
HDFs—human dermal fibroblasts
HECT—homologous to the E6AP carboxyl terminus
HGPS—Hutchinson–Gilford progeria syndrome
IAP—inhibitor of apoptosis
IF—intermediate filaments
INM—inner nuclear membrane
ISG15—interferon stimulated gene product 15
KAP1—Krüppel-associated box (KRAB)-associated protein 1
LAP2α—lamina-associated polypeptide 2α
LC3—microtubule-associated protein a light chain
MDM2—murine double minute 2
NAE—NEDD8 activating enzyme
NE—nuclear envelope
NEDD8—neural precursor cell expressed, developmentally down-regulated 8
NEDP1—NEDD8-specific protease 1 (also known as DEN1)
NLs—nuclear lamins
NLS—nuclear localization signal
PE—phosphatidylethanolamine
PIAS—protein inhibitor of activated STAT
PML*—*promyelocytic leukemia protein
pRb—retinoblastoma protein
PTMs—posttranslational modifications
RBR—RING-in-between-RING
RBX1/2—RING-box proteins 1 and 2
RING—really interesting new gene
Rmnd5a—required for meiotic nuclear division 5a
RNF—ring finger protein
ROC1—regulators of cullins 1
SCF—Skp1–Cullin–F-box proteins
SENP—Sentrin specific proteases
SIM—SUMO-interacting motif
SMURF2—Smad ubiquitin regulatory factor 2
SUMO—small ubiquitin-related modifier
TRIM—tripartite motif-containing protein
UB—ubiquitin
UBA—ubiquitin-activating enzyme
UBC—ubiquitin-conjugating enzyme
UBL—ubiquitin-like
UFC1—ubiquitin-fold modifier conjugating enzyme 1
UFL1—UFM1 specific ligase 1
UFM1—ubiquitin-fold modifier 1
ULMs—ubiquitin-like modifiers
ULPs—ubiquitin-like peptidases
URM1—ubiquitin-related modifier 1
USP—ubiquitin specific peptidase
